# Teenage Pregnancies and Childbirth Experience in Romania From the Midwives Point of View

**DOI:** 10.7759/cureus.13851

**Published:** 2021-03-12

**Authors:** Mihaela C Radu, Anca I Dumitrescu, Corneliu Zaharia, Calin Boeru, Melania E Pop-Tudose, Claudia F Iancu, Razvan D Chivu

**Affiliations:** 1 Birth Block, Obstetrics and Gynecology Hospital, Ploiesti, ROU; 2 Physics and Pharmaceutical Informatics, “Carol Davila” University of Medicine and Pharmacy, Bucharest, ROU; 3 Biophysics Laboratory, “Stefan S Nicolau” Institute of Virology, Bucharest, ROU; 4 Obstetrics and Gynaecology, “Carol Davila” University of Medicine and Pharmacy, Bucharest, ROU; 5 Nursing, “Carol Davila” University of Medicine and Pharmacy, Bucharest, ROU; 6 Public Health Sciences, “Carol Davila” University of Medicine and Pharmacy, Bucharest, ROU

**Keywords:** teenage pregnancy, midwives, homebirth

## Abstract

In Romania, in 2017, the infant mortality rate was eight per thousand; with 41,000 women who had no medical visits during pregnancy; 18,500 were teenagers.

Our objective was to analyze how many teen pregnancies were in an Obstetrics and Gynaecology Hospital from Romania over a two-year period and underline the role that midwives have in preventing teenage pregnancies.

A descriptive study of a group of 343 childbearing teenagers out of 7020 childbearing women who gave birth in 2017-2018 is presented. The teenagers were evaluated by age, the number of pregnancies, birth complications, way of delivery, and place of origin. The involvement of the midwife was highlighted.

From the total of 7020 analyzed cases, 4.8% (n=343) were teen pregnancies. Within this group, 4.37%(n=15)were already at the third birth and 89.79 (n=308)were un-investigated during the entire pregnancy. Sixty-eight point fifty-one percent (68.51%; n=235)of the teenagers gave birth with the aid of a midwife while 4.66 (n=16) gave birth with no medical attendance and experienced homebirth. All the teenagers that gave birth at home were from rural areas and not married.

High teenage pregnancy rates are determined in Romania by the low level of information about sexuality and family planning at young ages. The midwives have the ability to help to resolve these problems if they were more allowed to be involved in these programs, especially in rural areas.

## Introduction

In the European Union, according to data published by EUROSTAT, Romania and Bulgaria have the highest proportion of teenage pregnancies [1]. The Ministry of Health of Romania reports the highest rate of mortality for mother and child at birth from Europe and the highest rate of unattended pregnancies and teen pregnancies in the European Union [2].

In 2017, in Romania, the infant mortality rate was eight per thousand, almost twice the average rate of the European Union, according to the National Institute of Statistics from Romania. At the same time, there were 41,000 women who had no medical visits during pregnancy, some due to poverty and others due to difficult access to doctors' offices. Another 18,500 mothers were teenagers [3].

As a comparison, in the U.S., in 2017, a total of 194,377 babies were born in the 15-19 years age group, with a birth rate of 18.8 per 1,000 women for this age group. This is a drop of 7% from 2016 [4]. Birth rates fell 10% for women aged 15-17 years and 6% for women aged 18-19 years [5]. Still, in the U.S., the teen pregnancy rate is substantially higher than in other western industrialized nations [6] and racial/ethnic and geographic disparities in teen birth rates persist [7].

Teen pregnancy at a very early age, less than 15 years old, may bring for most adolescents insecurity, problems, fear, and many questions. It means problems at home with the parents, but, at the same time, problems with the father of the child. A study published by Sriyasak et al. shows that teenage mothers struggled with unexpected changes in their lives and teenage fathers experienced changed role expectations and responsibility [8-9].

The health hazards of the new-born from teen pregnancies may occur from the lack of a proper diet during pregnancy due to lack of health educational programs, especially in rural areas. In teenagers under 13 years old, many new-born health problems may occur due to the immaturity of the mother’s body. From a medical point of view, early adolescence is not anatomic-physiologically prepared to reproduce without risk. Romania holds first place in teen pregnancies in 2015 in Europe, according to a report published in 2018 [1], there were 9.78% of teen pregnancies from the total number of birth in 2015. So, of the teenagers between 15 and 19 years old, in Romania, every year, 5%-10% remain pregnant. The birth rate of these teenagers was constantly increasing according to earlier studies [10].

Recent studies showed that teen pregnancy is strongly influenced by childhood experiences in the family and social environment, especially those related to unstable parenting or early motherhood [11]. Women who grew up in poorer family environments (rural areas, large families, did not live with both parents, had poor education) show higher risks for early pregnancy and early non-marital birth [12]. Pregnancy in adolescence compromises women's educational prospects and economic opportunities, being a marker of such conditions, rather than a major cause of it [13].

Pregnancy and childbirth at an early age have major implications for the general and reproductive health of the teenager; these may either develop immediately after birth or after a certain period. In some cases, pregnancy at an early age is more complicated and involves certain risks, including during birth, which can affect the health of the mother or new-born. The experts paid attention to the psychological aspect of the phenomenon. As a teenager, the young girl does not have a coherent perception toward the new-born; they may not be responsible for him or take care of him properly. In many cases, childbearing teenagers neither have the necessary skills nor have they matured psychologically; the period of pregnancy and the one after the birth usually become strong psychological stress for these young childbearing women [14].

There are several aspects that may contribute to placing Romania in the first place in Europe regarding the number of births registered among minors [15] such as poverty and ignorance, the lack of a national sex education program, poor school education, and even promotion of sex as a way of life success. There is also the pressure of their entourage for having sex as early as possible, and this may be translated in the number of teen mothers that reached an average of 8,500 per year; 34 mothers out of a thousand had not reached the age of 18 years in 2015 according to United Nations International Children's Emergency Fund (UNICEF) studies [16].

In Romania, the Ministry of Health admits that these problems could be prevented with the help of midwives [3]. Currently, their role is only to assist the pregnancy within normal parameters, to monitor and assist the natural birth, to care for the mother and new-born for at least 28 days after birth, and to recognize any medical complications. But they could be more involved more in programs that help prevent unwanted pregnancies among teenagers because they have the necessary skills for that.

The World Health Organization (WHO) places the midwifery profession among the most important factors for the care of women of fertile age in good health [17].

In Romania, there are currently only 1000 licensed midwives, but they cannot be integrated into the care system due to the lack of legislation. So they function along with nurses in Obstetrical and Gynaecology Hospitals. Although in other countries of the European Union, the assistance provided by the midwife is a natural practice, in Romania, there are still no rules of practice, guides, and professional protocols that clearly regulate this profession, by harmonizing with the European legislation. In rural areas, midwives could make a real difference in Romania. The role of midwife should not be neglected, as she has the knowledge and the competence to perform several practical and educational tasks [8].

By this paper, the authors want to provide a general framework of teenage pregnancy and childbirth in the Hospital of Obstetrics and Gynaecology from Ploiesti, Romania, and to offer new data regarding teen pregnancy trends in Romania. Nowadays midwife practice is limited in Romania but midwives have the abilities that may help to decrease the number of teen pregnancies. We choose the Hospital of Obstetrics and Gynaecology from Ploiesti for our study. The hospital is in a country region that is around 60 km away from the capital of Romania, Bucharest, and is representative and similar to many other country hospitals from our country. The hospital serves not only the town of Ploiesti but also the rural surrounding area.

The present study aims to analyze how many teen pregnancies were in Ploiesti Obstetrics and Gynaecology Hospital during 2017 and 2018 and to expose the social factors that favor early childbirth in Romania. The midwives may have a big role, as they may influence and improve the social protection of young mothers and they can help to prevent teenage pregnancies, especially in rural communities.

## Materials and methods

We monitored all women who gave birth at Ploiești Obstetrics and Gynaecology Hospital over a two-year period (from January 2017 till December 2018). There were 7020 childbearing women who gave birth over the two-year period: 3580 in 2017 and 3440 in 2018. Our focus was on the 12-17 years old group. The age group was chosen according to the age at which a woman becomes officially an adult in Romania, which is 18 years old. The lower limit, 12 years of age, was given by the youngest teen pregnancy that occurred in our hospital. The teenagers were evaluated by age; number of pregnancies, birth complications, way of delivery, and place of origin.

All women who came to give birth signed an informed consent form regarding the medical procedures involved and the personal data management. The women included in our study had given informed consent to participate in the study. For all childbearing teenagers, consent to be involved in the study was also provided by their parents or legal guardians. Our research was carried out with the approval and in accordance with the guidelines of the local Ethics Committee.

The data were analyzed through the Microsoft Office package - Word and Excel (Microsoft Corporation, Redmond, WA). We calculated the p-value with the chi-square statistic test and considered a significant result when p <.05.

## Results

There were 4.8% (n=343) teen pregnancies among the total of 7020 analyzed childbearing women. We analyzed them by year in order to be able to evaluate the trend of changes. In 2017, 3580 women of different ages gave birth in the Obstetrics and Gynaecology Hospital of Ploiesti. From them, 182 were between 12 and 17 years old. 49% (n=90) of pregnant women were 17 years old, 31% (n=58) were 16 years old, and 20% (n=34) of them were in the 12-15 age group (23 were 15 years old, 10 were 14 years old, and one was 12 years old). There were no women under 12 years old who gave birth in our hospital in 2017 (Table 1). In 2018, 3440 women of different ages gave birth in the Obstetrics and Gynaecology Hospital of Ploiesti. From them, 161 were aged between 12 and 17 years. 43% (n=69) of pregnant women were 17 years old, 32% (n=52) were 16 years old and 25% (n=40) of them were in the 12-15 age group (31 were 15 years old, eight were 14 years old, and one was 13 years old). There were no women under 13 years old who gave birth in our hospital in 2018.

**Table 1:** Demographic characteristics of the studied group

Demographic data	N (%) 2017 182 (100%)	N (%) 2018 161 (100%)	P-value
Age			
12 years old	1 (0.549 %)	0	p is not applicable here
13 years old	0	1 (0.621 %)	
14 years old	10 (5.494 %)	8 (4. 968 %)	
15 years old	23 (12.637 %)	31 (19.254 %)	
16 years old	58 (31.868 %)	52 (32.298 %)	
17 years old	90 (49. 450 %)	69 (42.857 %)	
Social status			
Married	69(37.912%)	56(34.782%)	p=.54 (not significant at p < .05)
Single	113(62.087%)	105(65.217%)	
Occupation			
Student	4 (2.197%)	8 (4.969%)	p=.31 (not significant at p < .05)
Employee	1 (0.549%)	5(3.105%)	
Household	177 (97.252%)	148 (91.925%)	
Place of origin			
Urban	57(31.318%)	62(38.509%)	p=.16 (not significant at p < .05)
Rural	125(68.681%)	99(61.490%)	
Ethnicity			
Romanian	15 (8.241%)	29 (18.012%)	p=.006922 (is significant at p < .05)
Other ethnicity (Roma and Hungarian)	167 (91.758%)	132 (81.987%)	
Cultural influence/religion			
Orthodox	85 (46.703%)	76 (47.204%)	p=.99 (not significant at p < .05)
Atheist	14 (7.692%)	12 (7.453%)	
Other religion (Roman Catholics and Evangelists)	83 (45.604%)	73(45.341%)	

Although there were several ethnic origins in our studied groups besides Romanian ethnic origin (p=.006922), all teenagers had Romanian citizenship.

We noticed that the majority of the teenagers that gave birth came from rural areas in 2017, as well as in 2018, with a total of 65.59% (n=224) for the two investigated years.

A fact that was overwhelming was that some of the teenagers from the study gave birth to their second or even their third baby, p=.04 (Table 2). In 2017 there were four teenagers who gave birth for the third time, 21 who gave birth for the second time and the majority 86% (n=157) gave birth for the first time. The percentages were slightly different for 2018 when 77% (n=124) gave birth for the first time while 11 teenagers gave birth for the third time and 26 for the second time.

**Table 2:** Data regarding childbearing women and their pregnancies

Obstetrical data	N (%) 2017 182 (100%)	N (%) 2018 161 (100%)	p-value
Investigated current pregnancy			
Yes	22 (12.087 %)	13 (8.074%)	p=.22 (not significant at p < .05)
No	160 (87.912 %)	148 (91.925%)	
Way of giving birth			
In hospital with the midwife	120 (65.934%)	115 (71.428%)	p=.33 (not significant at p < .05)
C-section with the midwife present	51 (28.021%)	41 (25.465 %)	
Birth at home without medical supervision	11 (6.043%)	5 (3.105%)	
Number of babies at the current birth			
First baby	157 (86.263%)	124 (77.018%)	p=.04 ( is significant at p < .05)
Second baby	21 (11.538%)	26 (16.149%)	
Third baby	4 (2.197%)	11 (6.832%)	
Distribution of pregnancy-related diseases			
Infections	8 (4,395%)	14 (25,175%)	p is not applicable here
Obesity	3 (1,648%)	3 (1,863%)	
Pre-eclampsia	11 (6,043%)	14 (8,695%)	
Thrombocitopenia	2 (1,098%)	1 (0,621%)	
Rh incompatibillity	6 (3,296%)	5 (3,105%)	
Anemia	11 (6,043%)	9 (5.590%)	
Uterus with scars	8 (4.395%)	6 (3.726%)	
Disproportion between the fetal head and the mother’s pelvis	10 (5.494%)	11 (6.832%)	
Distribution of after-birth complications			
Only for women who gave birth naturally	N (%) 2017 131 (100%)	N (%)2018 120 (100%)	p-value
Episiotomy	102 (77.862%)	74 (61.666%)	p=.012 (is significant at p < .05)
Hematoma	5 (3.816%)	8 (6.666%)	
Manual control	25 (19.083%)	45 (37.5%)	
Lacerations of the birth canal	51 (38.931%)	48 (40 %)	

The majority of teen pregnancies were not monitored during the pregnancy, the adolescents came to the hospital only to give birth, and they had no investigation at all during the entire pregnancy. So in 2017, there were 88% (n=160) un-investigated teen births and in 2018, there were 92% (n=148) un-investigated teen births.

According to the way of delivery, we had three situations: the teenagers that presented themselves to the hospital and gave birth with the aid of a midwife; in 2017, there were 120 teenagers, and in 2018 there were 115.

The second situation was delivery through a C-section performed by a gynecologist with the aid of a midwife. In this situation, the midwife was responsible for the new-born; she took the child from the gynecologist, dried him, stimulated him if needed, and then took care of the umbilical cord. There were 51 adolescents in 2017 that gave birth through C-section and 41 in 2018. The third situation was when the teenager gave birth at home. This was the most unfortunate situation. In Romania, the legislation does not allow women to give birth at home. Giving birth at home alone, without medical care, is dangerous and in all cases, this happens due to ignorance and lack of information about the pregnancy. In this situation, both the teenager and the new-born were facing potential complications and medical problems because they were not supported or helped in any way throughout the childbirth. After the childbirth, the teenagers were taken to the hospital by a hospital rescue car. At the hospital, a midwife evaluated them and the new-born. Because of the lack of a specialist during childbirth, the new-born could not receive an Apgar score. There were 11 teenagers that gave birth at home in 2017 and five in 2018. Out of them, only four were attending school, the rest had dropped out. There were no premature new-borns in 2017, and there were three premature new-born babies in 2018. In 2017, the median age of the teenagers that gave birth at home was 17 years old, with limits from 14 to 17 years old. In 2018, the median age was 16 years old, with limits between 15 and 16 years old.

Surprisingly, in 2018, from the five teenagers that gave birth at home without medical care, three were at the second birth already, and they all were only 15 years old. So they had a history of teen pregnancies that may have been prevented, in our opinion, with the aid of the midwife if a legal frame for this would have existed.

Although we are discussing the 12-17 age group, we noticed that almost a third of the teenagers that gave birth in the study presented diseases associated with pregnancy, previously undiagnosed and untreated: 59 in 2017 and another 63 in 2018.

Some of the teenagers that gave birth in the hospital presented complications during childbirth, p=.012. The most frequent disturbing complication was lacerations of the birth canal; 28.86% of the teenagers (n=99) presented with it. Episiotomy was performed for 51.31% (n=176) of the teenagers. Another intervention was manual control for the removal of fragments of the placenta. All these interventions were performed by the midwife under the gynecologist's supervision. Three point seventy-nine percent (3.79%) (n=13) of the adolescents presented hematoma as a birth complication.

## Discussion

Around the world, teenage pregnancies are more likely to occur in marginalized communities, commonly driven by lack of education, unemployment, gender inequality, lack of sexual and reproductive health services, and the low status of women and girls [16].

The latest data published for Romania was in 2018 in the Complete End of Childhood Index 2018 where the adolescent birth rate (births per 1,000 girls aged 15-19) from 2015 was 34 per 1,000 birth.

Comparing the two analyzed years from our study, we can see a slight decrease in the birth rate of teen pregnancies in 2018 from 50.83 per 1,000 births in 2017 to 46.80 per 1,000 births in 2018. But this data cannot be accounted as statistically significant for the country because the data come only from a single hospital. Nevertheless, the number of teen pregnancies from 1,000 births is higher than in the US, where, for example, in 2017, a birth rate of 18.8 per 1,000 women was published [4], and we will probably maintain our first position in Europe when new data will be released. In our study, we noticed that according to age, of most of the teenagers that gave birth, 78.42% (n=269) were at least 16 years old.

A study of the WHO Global Health Observatory Data Repository from 2017 had shown that the girls that have poor social conditions have about three times as many births as the wealthiest [17].

In our study, all teenagers had poor social conditions and poor education. Although 2018 was a year with fewer teen pregnancies in our hospital, the percentages of adolescents that gave birth for the second or even for the third time at such an early age was similar to the one encountered in 2017. In our study, there were 15 teenagers that gave birth for the third time and 47 that gave birth for the second time, that is 18.07% (n=62) of teenagers that had more than one pregnancy until the age of adulthood.

The organization "Save the Children" published a report in 2018 "The many faces of exclusion," where they explained that teenage pregnancy can have negative economic consequences on girls and their families and communities, and costs countries billions of dollars each year. Because of their lower educational status, many teen mothers have fewer skills and opportunities for employment, often perpetuating cycles of poverty. Nationally, this can also have an economic cost, with countries losing out on the income that young women would have earned over their lifetimes in case they had not early pregnancies [16].

In Romania, the teenagers that gave birth were mainly unemployed: in 2017, there were 97.25% (n=177) unemployed teenagers and in 2018, there were 91.92% (n=148) unemployed and only a few attended schools.

According to Romanian law in 2019 (law 287/2009), a marriage can take place if future spouses are 18 years of age, but for good reasons, the minor who has reached the age of 16 years may marry on the basis of a medical opinion, with the consent of his parents or, as the case may be, of the guardian and with the authorization of the guardianship court in which the minor has his domicile [18].

In our study, during all analyzed periods, we found that 97.09 (n=333) teenagers were not married and 89.79% (n=308) did not seek professional health care services at all during their pregnancy, a fact that confirms the Indian study by Paul et al., which revealed that women who married at <18 years were significantly less likely to use maternal health care services than those married at ≥18 years even after accounting for the socio-economic and demographic characteristics of women [19].

In the studied group aged 12-17, we noticed that almost a third (n=118) of the teenagers that gave birth presented diseases associated with pregnancy that were previously undiagnosed and untreated. Of them, 18.64% (n=22) had infectious diseases. In this context, special attention should be given to sexually transmitted diseases that can infect the young pregnant woman and then can be transmitted to the new-born, affecting, in this way, the life of the future child [20-21].

The majority of the teenagers chose to give birth in the hospital with the aid of the midwife (68.51%, n=235). There were only 4.66% (n=16) of teenagers that experienced homebirth. All of them were from rural areas and were not married. We identified them as being of Rroma ethnicity and Orthodox religion. We could not associate with our study any of the themes from the literature on unassisted childbirth and high-risk homebirth such as (1) resisting the biomedical model of birth by trusting intuition, (2) challenging the dominant discourse on risk by considering the hospital as a dangerous place, (3) feeling that true autonomous choice is only possible at home, (4) perceiving birth as an intimate or religious experience, and (5) taking responsibility as a reflection of true control over decision-making [22]. The fact that, out of the 16 teenagers that experienced homebirth, only four attended school and the rest dropped out confirms that poor education was, in fact, the reason for their choice.

As in other countries from the world, where homebirth was not integrated into the maternity care system, in Romania, the midwife is needed to attend high-risk homebirth. Munro et al., in 2013, have shown that there are significant barriers in the collaboration between care provider groups, including skill sets, professional orientation, and funding models [23]. The rural communities, such as those encountered in our study, and the limited health resources may benefit from the presence of the midwife, especially in teenage homebirth. A midwife has the skills for providing care and education in pregnancy, and a legal framework would allow her to exercise it in Romania too. The education that the midwife can provide is important and could cover many aspects, from planning the pregnancy to infectious diseases that may be transmitted from mother to child and could be prevented through vaccination or other methods [24-29].

## Conclusions

Our study showed that most of the teenagers that experienced childbirth had a poor education. We believe that the midwife is very important and may help and improve medical care, especially in rural communities. Teenagers may benefit from the midwives' experience and may be advised to understand the danger of birthing outside the system in Romania, where there is no legal background for homebirth. Particular importance should be given to the sexual and reproductive health of teenagers. Midwives can help and support these educational programs if they could act as individual entities and the law would support them.

Improving the health of teenagers, increasing their security and well-being presents a complex approach that requires the efforts of several sectors and social institutions such as educational institutions, health and social care institutions, local government, youth organizations, the church, and the media, including parents and families. We consider that the development of national programs and strategies in the field of sexual and reproductive health with a strong accent on family planning is needed. Midwives may hold a key role in education and prevention. Family planning may act as an intervention, including services for the prevention of unwanted pregnancy, reduction of sexually transmitted infections, aiming at reducing the morbidity and mortality of infants and mothers, preventing/treating infertility, preventing abuse and sexual violence. Collaborations between specialists, including midwives in all these programs, or creating a multidisciplinary network could be of great help. To conclude, high teenage pregnancy rates are determined by the low level of information about sexuality and family planning, the insufficiency of sexual education programs, and the lack of structures and laws that involve midwives in the programs.
